# Dynamic Variation of Secondary Metabolites from *Polygonatum cyrtonema* Hua Rhizomes During Repeated Steaming–Drying Processes

**DOI:** 10.3390/molecules30091923

**Published:** 2025-04-25

**Authors:** Shuzhen Wang, Feng He, Ruibin Hu, Xuchun Wan, Wei Wu, Lei Zhang, Chi-Tang Ho, Shiming Li

**Affiliations:** 1 Lishizhen College of Traditional Chinese Medicine, Huanggang Normal University, Huanggang 438000, Chinahuruibin@163.com (R.H.); wanxuchun2008@163.com (X.W.); woowe97417@126.com (W.W.);; 2 Hubei Key Laboratory of Germplasm Improvement and Utilization of Genuine Medicinal Materials in Dabie Mountains, Huanggang 438000, China; 3 Department of Food Science, Rutgers University, New Brunswick, NJ 08901, USA

**Keywords:** Polygonati Rhizoma, *Polygonatum cyrtonema*, homology of medicine and food, health-promoting activities, metabolomics, postharvest processing

## Abstract

Polygonati Rhizoma, widely used as a traditional functional food and herbal medicine, is well known for its health-promoting activities after the process of “nine cycles of steaming-drying”. Based on UPLC-MS/MS, 1369 secondary metabolites were identified in *P. cyrtonema* rhizomes, mainly alkaloids, amino acids and derivatives, flavonoids, organic acids, phenolic acids, and saccharides. The *P. cyrtonema* rhizomes were rich in xylose, arabinose, glucose, sorbose, mannose, galactose, rhamnose, inositol, fucose, sedoheptulose, phosphorylated monosaccharides, sugar acid, and sugar alcohols. Particularly, 23 types of modifications were detected for amino acids, while the most frequent modifications were acetylation, methylation (nono-, di-, and tri-), cyclo-, homo-, and hydroxylation. Based on the metabolic profile, samples from the third cycle (Tre-3) and the sixth cycle (Tre-6) were firstly clustered together due to similar metabolites and then grouped with samples from the ninth cycle (Tre-9). Differentially accumulated metabolites were mainly enriched in “Metabolic pathways”, “Biosynthesis of cofactors”, “Biosynthesis of secondary metabolites”, “Flavonoid biosynthesis”, “Purine metabolism”, “ABC transporters”, “Biosynthesis of amino acids”, and “Nucleotide metabolism” pathways. During repeated steaming–drying processes, 39 metabolites occurred, including alkaloids, amino acids and derivatives, flavonoids, lignans and coumarins, lipids, nucleotides and derivatives, organic acids, phenolic acids, and terpenoids. This research will provide a critical scientific basis for postharvest processing of *P. cyrtonema* rhizomes.

## 1. Introduction

Polygonati Rhizoma, prepared from rhizomes of Polygonatum cyrtonema Hua, *P. kingianum* Coll. et Hemsl, and *P. sibiricum* Redoute through the process of “nine cycles of steaming-drying process”, has been widely used for thousands of years in China and Southeast Asia [1,2,3]. An example of medicine and food homology, Polygonati Rhizoma is effective in treating diabetes mellitus, influenza, fevers, backache, sexual dysfunction, inappetence, indigestion, mastitis, fatigue, feebleness, dizziness, and lung trouble [4,5]. After repeated steaming–drying processes, Polygonati Rhizoma is broadly used as a main component in functional food and the pharmaceutical industry due to its health-promoting activity, sweet fragrance, and excellent taste [6,7,8]. In particular, Rhizoma Polygonati is often processed into functional wines, teas, and botanical beverages, as well as medicinal diets [9,10].

*P. cyrtonema*, a typical member of the genus Polygonatum (Asparagaceae) and a rhizome propagating herb, is endemic to China, especially the central and eastern regions [4]. Compared with *P. sibiricum* and *P. kingianum*, the dried roots of *P. cyrtonema* are optimal in quality and yield [11]. The first monograph book on pharmacology in China, Shennong Bencao Jing, classified *P. cyrtonema* as a top-grade herb [9]. According to growing characteristics, the perennial *P. cyrtonema* is often growing and cultivated under forest cover [12]. The Dabie Mountains of central China are the main natural distribution and artificial cultivation base for *P. cyrtonema*. In previous studies, various flavonoids, saponins, polysaccharides, and alkaloids with health-promoting properties have been isolated from *P. cyrtonema* rhizomes [13,14,15]. Particularly, the flavonoids and other secondary metabolites exhibit remarkable antioxidant, anti-tumor, and immunomodulatory effects [16]. Based on RNA-seq from fruits, leaves, roots, stems, and rhizomes of three-year-old P. cyrtonema plants, 379 differentially expressed genes (DEGs) were obtained, while transcripts of flavonoid-biosynthesis-related DEGs were principally enriched in rhizomes [16]. Transcription factor families *MYB*, *WRKY*, and *AP2/ERF* are positively correlated with rhizome flavonoid accumulation, while the differentially expressed genes of *BZIP1*, *C3H31*, *ERF114*, and *DREB21* also accompany the development of *P. cyrtonema* rhizomes [16]. Flavonoids and polysaccharides are the main medicinal ingredients of *P. cyrtonema* rhizomes and serve as important indicators of the quality of *P. cyrtonema* rhizomes [17].

However, the dynamic variation of secondary metabolites in *P. cyrtonema* rhizomes during repeated steaming–drying processes has not been systematic studied. Furthermore, the necessity of nine cycles should also be confirmed. Metabolomics, the global analysis of large numbers of certain cellular metabolites, shows great potential in elucidating the dynamic variation of secondary metabolites [18,19,20]. In this study, the dynamic changes of *P. cyrtonema* rhizome metabolites processed through different periods of steaming and drying were deeply analyzed through UPLC-MS/MS, aiming to supply a critical scientific basis for postharvest processing.

## 2. Results

### 2.1. Metabolite Identification in P. cyrtonema Rhizomes

**Figure 1:**
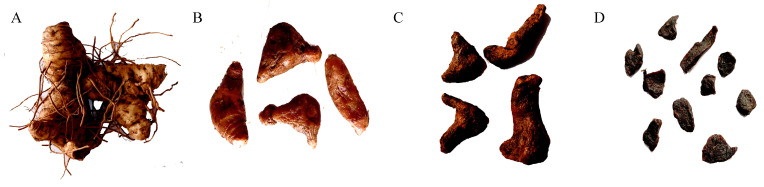
*P. cyrtonema* rhizomes with different steaming–drying processes: (**A**) 0 cycles; (**B**) 3 cycles; (**C**) 6 cycles; and (**D**) 9 cycles.

**Figure 2:**
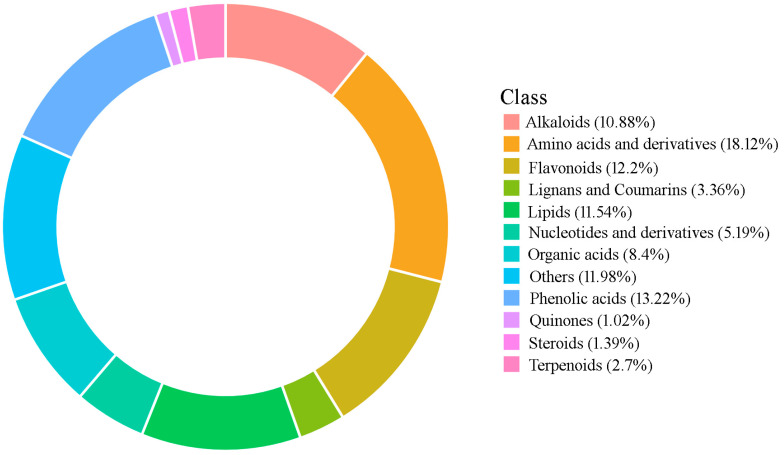
Ring chart of categorization of metabolites identified in *P. cyrtonema* rhizomes.

In order to ensure stability, 12 libraries for 4 groups of samples were constructed, including Tre-0, Tre-3, Tre-6, and Tre-9, which were subjected to zero, three, six, and nine cycles of steaming–drying, respectively (Figure 1). Based on UPLC-ESI-MS/MS analysis, a list of 1369 secondary metabolites were successfully identified, including 149 alkaloids, 167 flavonoids, 46 lignans and coumarins, 158 lipids, 14 quinones, 19 steroids, 37 terpenoids, 248 amino acids and derivatives, 71 nucleotide compounds, 115 organic acids, and 181 phenolic acids, as well as 164 other compounds including alcohols, aldehydes, chromones, ketones, saccharides, and vitamins (Table 1 and Table S1, Figure 2).

Alkaloids, flavonoids, lignans, and coumarins have been reported to exhibit monoamine oxidase inhibitory activity, antioxidative activity, and antifungal activity, which enable hosts to resist adverse environments [20,21]. In relation to Polygonati Rhizoma, alkaloids, phenolamines, plumeranes, quinoline alkaloids, pyridine alkaloids, pyrrole alkaloids, piperidine alkaloids, isoquinoline alkaloids, tropan alkaloids, and benzylphenylethylamine alkaloids were found to constitute the alkaloids type, which might play important roles in health-promoting activities (Table 1). Furthermore, flavones, flavanones, flavonols, chalcones, isoflavones, flavanols, and flavanonols were found to be the main types of flavonoids, and 11 coumarins and 35 lignans were found to exist in Polygonati Rhizoma. In addition, 80 free fatty acids, 10 glycerol esters, 29 lysophosphatidylcholines (LPC), 31 lysophosphatidylethanolamines (LPE), 1 phosphocholines (PC), and 7 sphingolipids were screened for the lipids class. Regarding the steroids class, 6 steroids and 13 steroidal saponins were found. The terpenoids class consisted of 15 ditepenoids, 10 monoterpenoids, 4 sesquiterpenoids, 7 triterpenoids, and 1 triterpene saponin (Table 1 and Table S1).

The 20 natural amino acids, including Ala, Asn, Asp, Arg, Cys, Gly, Glu, Gln, His, Ile, Leu, Lys, Met, Phe, Pro, Ser, Thr, Tyr, Trp, and Val, were all present in *P. cyrtonema* rhizomes (Table S1). In addition, a number of non-protein amino acids were also found, such as D-Leu, allysine, jasmonoyl-L-isoleucine, omega-aminoarginine, pyroglutamic acid, palmitoylglycine, L-Homocitrulline, and (3-(carboxyamino)-2-methylpropanoyl) phenylalanine. In total, 22 types of modifications in amino acids were detected, especially acetylation, cyclo-, hydroxylation, methylation (nono-, di-, and tri-), and homo-modification (Table 2). Furthermore, Tyr, Pro, and Gly all had six types of modification, followed by Arg and Cys with five types each. Besides cyclization, Tyr could be modified by acetyl-, hydroxyl-, oxo-, nitro-, and phosphorylation. Regarding Pro, the six types of modifications were cyclization, hydroxylation, monomethylation, homo-, oxo-, and propylation.

In total, nine vitamins and corresponding derivatives were obtained, including both fat-soluble vitamins (vitamin E and vitamin K) and water-soluble vitamins (vitamin C and vitamin B family) (Table S2). Both vitamin K1 and vitamin K2 were present in *P. cyrtonema* rhizomes. Regarding the vitamin B family, five members were found, including vitamins B2, B3, B5, B6, and B13. A total of four and five derivatives were identified for vitamin B3 and vitamin B5, respectively. However, vitamin B2, vitamin B5, and vitamin B13 all had one derivative.

Among saccharides, 46 monosaccharides, 14 disaccharides, 9 trisaccharides, 4 tetrasaccharides, and 1 pentasaccharide were found (Table 3). In regard to monosaccharides, the carbon numbers ranged from 3 to 7. The *P. cyrtonema* rhizomes were rich in xylose, arabinose, glucose, sorbose, mannose, galactose, rhamnose, inositol, fucose, and sedoheptulose. In particular, 11 phosphorylated monosaccharides were identified, especially six-carbon monosaccharides. Moreover, 8 monosaccharides were oxidized to sugar acid, while 9 monosaccharides were deoxidized to sugar alcohols. In addition, 3 monosaccharides had lactonization modification. For 1,5-anhydro-d-glucitol and 1,6-anhydro-*β*-d-glucose, dehydration occurred. Amination took part in both D-glucosamine and *N*-acetyl-d-galactosamine. The glucopyranose was esterified by 6-hydroxydecanoate. In related to disaccharides, phosphorylated derivatives were found.

### 2.2. Variation Trends of Metabolites During Repeated Cycles of Steaming and Drying

The majority of secondary metabolites were present in all four groups of samples, while the contents for each metabolite varied in different samples. According to the variation trends of the contents, the 1369 metabolites could be divided into 6 types, including ‘Type I’ (the contents gradually decreased during steaming–drying processes), ‘Type II’ (the contents firstly decreased then increased during steaming–drying processes), ‘Type III’ (the contents firstly decreased, then increased, and finally decreased during steaming–drying processes), ‘Type IV’ (the contents gradually increased during steaming–drying processes), ‘Type V’ (the contents firstly increased then decreased during steaming–drying processes), and ‘Type VI’ (the contents firstly increased, then decreased, and finally increased during steaming–drying processes) (Tables S3 and S4). All the 186 metabolites of ‘Type I’ had the highest contents in Tre-0 samples, but gradually degraded during repeated processing, represented by gluconic acid, (+)-7-iso-jasmonic acid, sorbose, homogentisic acid, verbascose, 4-methylbenzaldehyde, L-citramalic acid, 3-hydroxyglutaric acid, pentadecanoic acid, palmitic amide, cajanin, 2-phenylethylamine, cinnamoyltyramine, and polygonatoside D (Table S4). In relation to ‘Type II’, 86 metabolites were screened, with the lowest values in Tre-3/Tre-6 samples and the highest values in Tre-9 samples (narirutin, citric acid glucoside, isoleucylleucine, riboprine, Gly-Pro-Glu, cis-aconitic acid, and Trp-Pro-Asp). In addition, the variation trends of 109 metabolites were defined as ‘Type III’, with the highest values in Tre-0/Tre-6 and lowest values in Tre-3/Tre-9 samples (benzoylformic acid, nicotinamide, glucosyloxybenzoic acid, β-sitosterol, roseoside, eriodictyol, and 6-hydroxyhexanoic acid). ‘Type IV’ contained 171 metabolites, mainly 8-hydroxyquinoline, D-mannitol, 3-aminosalicylic acid, L-valine, agmatine, and δ-tocopherol. The majority of the metabolites belonged to ‘Type V’ (708), with the highest values in Tre-3 or Tre-6 samples, mainly represented by hexadecanedioic acid, 4-hydroxysphinganine, majoroside, LysoPC 16:0, pimaric acid, and narcissin. For the 109 metabolites belonging to ‘Type VI’, the highest values were found in Tre-3 or Tre-9 samples, mainly comprising phylloquinone, disporopsin, eucommiol, 4-oxopentanoic acid, and p-coumaric acid.

Compared with the treated Rhizoma Polygonati samples (‘Tre-3’, ‘Tre-6’, and ‘Tre-9’), 39 secondary metabolites could not be detected in ‘Tre-0’ sample, including alkaloids (2), amino acids and derivatives (6), flavonoids (7), lignans and coumarins (2), lipids (6), nucleotides and derivatives (3), organic acids (2), others (5), phenolic acids (5), and terpenoid (1) (Table S5). In particular, the representative substances were 1,14-tetradecanedioic acid, 1-α-linolenoyl-glycerol, ricinoleic acid, 8-hydroxyquinoline, 5-O-p-coumaroylquinic acid O-glucoside, D-mannitol, vitamin B2, citric acid-1-O-diglucoside, kaurenoic acid, 6,7,8-tetrahydroxy-5-methoxyflavone, LysoPC 16:0, methoxyapigenin, agmatine, LysoPC 18:3, maltotriose, methylophiopogonanone B, 6-aminocaproic acid, Phe-Ala-Gly, chrysoeriol-7-O-glucoside, N-cis-feruloyl-3′-O-methyldopamine, and narcissin. Furthermore, 4 secondary metabolites could not be detected in Tre-3 samples, including 1 flavone (α-hydroxycinnamic acid), 1 flavonol (4-[3-(4,8-dimethylnona-3,7-dienyl)-3-methyloxiran-2-yl]butan-2-one), 1 organic acid (riboprine), and azetidine-2-carboxylic acid. Additionally, 5 secondary metabolites were totally destroyed in Tre-6 samples, including 1 calyxanthone, α-hydroxycinnamic acid, 4-*O*-β-d-glucopyranosylferulic acid, and 2 organic acids. For Tre-9 samples, 9 secondary metabolites were destroyed, including 1 alkaloid, 4 flavonoids, 1 azetidine-2-carboxylic acid, and 3 phenolic acids.

Among the 1,369 metabolites, 314 (22.9%), 352 (25.7%), 418 (30.5%), and 285 (20.8%) compounds had the highest contents in the ‘Tre-0’ (Table S6A), ‘Tre-3’ (Table S6B), ‘Tre-6’ (Table S6C), and ‘Tre-9’ samples (Table S6D), respectively. In particular, alkaloids and phenolamine with the highest contents were mainly found in ‘Tre-0’ or ‘Tre-3’ samples (Table 4). Amino acids and derivatives with the highest contents were almost evenly distributed among four Rhizoma Polygonati samples. Among the 63 flavones, 44 were concentrated in ‘Tre-6’ (19) and ‘Tre-9’ (25) samples.

Furthermore, free fatty acids, glycerol ester, LPC, and LPE with the highest contents were mainly found in ‘Tre-3’ and ‘Tre-6’ samples. In addition, nucleotides and phenolic acids with the highest contents were mainly found in ‘Tre-3’ samples. Anthraquinone and ditepenoids with the highest contents were mainly present in ‘Tre-6’ samples. α-hydroxycinnamic acid and 4-[3-(4,8-dimethylnona-3,7-dienyl)-3-methyloxiran-2-yl]butan-2-one were present only in ‘Tre-0’ samples; they were destroyed through repeated steaming–drying processes and could not be detected in ‘Tre-3’, ‘Tre-6’, and ‘Tre-9’ samples. Azetidine-2-carboxylic acid was mainly present in ‘Tre-0’ samples, but was destroyed after three cycles of steaming and drying. The contents of 4-hydroxysphinganine were the highest in ‘Tre-3’ samples, but could not be detected in ‘Tre-9’ samples. In particular, *O*-β-d-glucopyranosylferulic acid was mainly found in ‘Tre-0’ samples.

### 2.3. Screening of Differentially Accumulated Metabolites

#### 2.3.1. ‘Tre-0’ Sample vs. ‘Tre-3’ Sample

**Figure 3:**
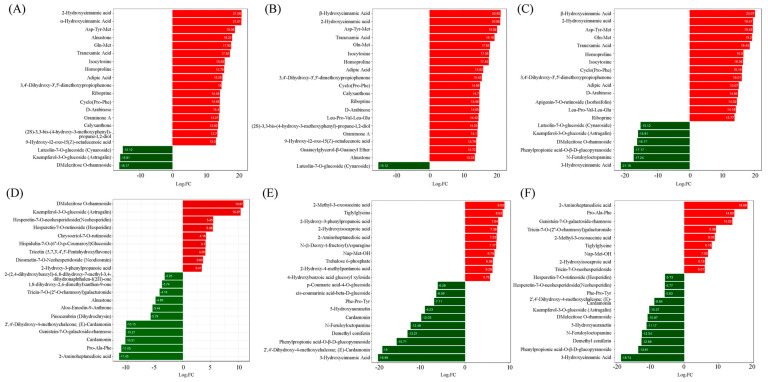
Bar chart of metabolites based on difference multiplier, with the X-axis representing log2FC values of metabolites and the Y-axis referring to differentially accumulated metabolites. The red and green colors refer to upregulation and downregulation, respectively. (**A**–**F**) referred to the ‘Tre-0’ Sample vs. ‘Tre-3’ Sample, ‘Tre-0’ Sample vs. ‘Tre-6’ Sample, ‘Tre-0’ Sample vs. ‘Tre-9’ Sample, ‘Tre-3’ Sample vs. ‘Tre-6’ Sample, ‘Tre-3’ Sample vs. ‘Tre-9’ Sample, and ‘Tre-6’ Sample vs. ‘Tre-6’ Sample, respectively.

Compared with ‘Tre-0’ samples, 825 metabolites were differentially accumulated in ‘Tre-3’ samples, including 662 upregulated and 163 downregulated metabolites. These differentially accumulated metabolites included alkaloids, amino acids and derivatives, flavonoids, lignans and coumarins, lipids, nucleotides and derivatives, organic acids, phenolic acids, quinones, steroids, terpenoids, and others (Tables S7 and S8). Among all these differentially accumulated metabolites, 355 metabolites could be enriched into 92 different KEGG pathways (Table S9A). The main differently accumulated metabolites were 2-hydroxycinnamic acid, α-hydroxycinnamic acid, Asp-Tyr-Met, DMelezitose O-rhamnoside, and kaempferol-3-*O*-glucoside (Figure 3A). “Metabolic pathways” (ko01100), “Biosynthesis of cofactors” (ko01240), “Biosynthesis of secondary metabolites” (ko01110), “ABC transporters” (ko02010), “Biosynthesis of amino acids” (ko01230), and “Nucleotide metabolism” (ko01232) were the main enriched KEGG pathways. In particular, “Linoleic acid metabolism”, “Nucleotide metabolism”, “Purine metabolism”, and “Pyrimidine metabolism” were the main enriched metabolism pathways (Figure S1A).

#### 2.3.2. ‘Tre-0’ Sample vs. ‘Tre-6’ Sample

Compared with ‘Tre-0’ samples, a list of 862 metabolites was differentially accumulated in ‘Tre-6’ samples, containing 674 upregulated and 188 downregulated metabolites (Tables S7 and S8). These differentially accumulated metabolites could be divided into 12 classes, including alkaloids, amino acids and derivatives, flavonoids, lignans and coumarins, lipids (only 126 upregulated), nucleotides and derivatives, organic acids, phenolic acids, quinones, steroids, terpenoids, and others. The main differently accumulated metabolites were α-hydroxycinnamic acid, 2-hydroxycinnamic acid, Asp-Tyr-Met, D-Melezitose O-rhamnoside, and Luteolin-7-*O*-glucoside (Figure 3B). For these differentially accumulated metabolites, 206 metabolites could be enriched into 91 different KEGG pathways (Table S9B). The typical KEGG pathways were “Metabolic pathways” (ko01100), “Biosynthesis of cofactors” (ko01240), “ABC transporters” (ko02010), and “Nucleotide metabolism” (ko01232), while the numbers of significantly enriched metabolites were 157, 72, 30, 21, and 19, respectively. “Biosynthesis of cofactors”, “Linoleic acid metabolism”, and “Nucleotide metabolism” were the main enriched metabolism pathways (Figure S1B).

#### 2.3.3. ‘Tre-0’ Sample vs. ‘Tre-9’ Sample

Compared with ‘Tre-0’ samples, 916 metabolites were differentially accumulated in ‘Tre-9’ samples, consisting of 645 upregulated and 271 downregulated metabolites (Tables S7 and S8). These differentially accumulated metabolites included alkaloids, amino acids and derivatives, flavonoids, lignans and coumarins, lipids, nucleotides and derivatives, organic acids, phenolic acids, quinones, steroids, terpenoids, and others. For the terpenoids class, all five subclasses were upregulated, while only the contents of monoterpenoids and terpene were downregulated. Among all differentially accumulated metabolites, 221 significantly differently accumulated metabolites could be enriched into 93 different KEGG pathways (Table S9C). The main differently accumulated metabolites were α-hydroxycinnamic acid, 2-hydroxycinnamic acid, Asp-Tyr-Met, and 3-hydroxycinnamic acid (Figure 3C). The typical KEGG pathways were “Metabolic pathways” (ko01100), “Biosynthesis of cofactors” (ko01240), “ABC transporters” (ko02010), and “Biosynthesis of amino acids” (ko01230), while the numbers of significantly enriched metabolites were 173, 36, 31, and 26, respectively. “Biosynthesis of cofactors”, “ABC transporters”, and “2-Oxocarboxylic acid metabolism” were the main enriched metabolism pathways (Figure S1C).

#### 2.3.4. ‘Tre-3’ Sample vs. ‘Tre-6’ Sample

Compared with ‘Tre-3’ samples, 255 metabolites were differentially accumulated in ‘Tre-6’ samples, including 124 upregulated and 131 downregulated metabolites (Tables S7 and S8). These differentially accumulated metabolites could be divided into 12 classes, mainly including alkaloids, amino acids and derivatives, flavonoids, lignans and coumarins, lipids, nucleotides and derivatives, organic acids, phenolic acids, quinones (only 4 downregulated), steroids (only 5 downregulated), and others. For the lipids class, 2 upregulated subclasses were detected, namely LPC and PC, while only the LPE subclass was downregulated. For the terpenoids class, the upregulated subclass was ditepenoids, while the downregulated subclass was monoterpenoids. The main differently accumulated metabolites were D-melezitose O-rhamnoside, kaempferol-3-O-glucoside, 2-aminoheptanedioic acid, and Pro-Ala-Phe (Figure 3D). Among differentially accumulated metabolites, 353 metabolites could be enriched into 60 different KEGG pathways (Table S9D). In particular, 55 metabolites were significantly enriched. The typical KEGG pathways were “Metabolic pathways” (ko01100), “Biosynthesis of cofactors” (ko01240), “Flavonoid biosynthesis” (ko00941), and “ABC transporters” (ko02010), while the numbers of significantly enriched metabolites were 41, 20, 8, and 7, respectively. Furthermore, “Flavonoid biosynthesis”, “Arginine biosynthesis”, “Purine metabolism”, and “Ascorbate and aldarate metabolism” were the main enriched metabolism pathways (Figure S1D).

#### 2.3.5. ‘Tre-3’ Sample vs. ‘Tre-9’ Sample

Compared with ‘Tre-3’ samples, 532 metabolites were differentially accumulated in ‘Tre-9’ samples, including 170 upregulated and 362 downregulated metabolites (Tables S7 and S8). For the lipids class, only the subclass PC was upregulated, while 4 subclasses were downregulated, namely free fatty acids, LPC, LPE, and sphingolipids. For the terpenoids class, the upregulated subclasses were ditepenoids and monoterpenoids, while the downregulated subclasses were monoterpenoids and terpene. A list of 105 metabolites were significantly enriched, mainly in “Metabolic pathways” (ko01100), “Biosynthesis of secondary metabolites” (ko01110), “Purine metabolism” (ko00230), “ABC transporters” (ko02010), and “Biosynthesis of cofactors” (ko01240) (Table S9E). In particular, “Biosynthesis of secondary metabolites” and “Nucleotide metabolism” were the main enriched metabolism pathways, with the main differently accumulated metabolites being 2-methyl-3-oxosuccinic acid, triglylglycine, and 3-hydroxycinnamic acid (Figure 3E and Figure S1E).

#### 2.3.6. ‘Tre-6’ Sample vs. ‘Tre-9’ Sample

Compared with ‘Tre-6’ samples, 420 metabolites were differentially accumulated in ‘Tre-9’ samples, including 121 upregulated and 299 downregulated metabolites (Tables S7 and S8). These differentially accumulated metabolites could be divided into 12 classes, including alkaloids, amino acids and derivatives, flavonoids, lignans and coumarins, lipids, nucleotides and derivatives, organic acids, phenolic acids, quinones, steroids (9 downregulated), terpenoids, and others. For the alkaloids class, only one alkaloid and one plumerane were upregulated. For the flavonoids class, 3 subclasses were upregulated, namely flavones, flavonols, and isoflavones. For the lipids class, 5 subclasses were detected, namely free fatty acids, glycerol ester, LPC, LPE, and sphingolipids. For the terpenoids class, the upregulated subclasses were ditepenoids and monoterpenoids, while the downregulated subclasses were ditepenoids, monoterpenoids, and terpene. In total, 64 KEGG pathways were enriched (Table S9F). The typical KEGG pathways were “Metabolic pathways” (ko01100), “Biosynthesis of secondary metabolites” (ko01110), “ABC transporters” (ko02010), and “Biosynthesis of cofactors” (ko01240), while the numbers of significantly enriched metabolites were 64, 36, 14, and 12, respectively. “Biosynthesis of secondary metabolites”, “ABC transporters”, “Purine metabolism”, and “Galactose metabolism” were the typical pathways, with the main differently accumulated metabolites being 2-aminoheptanedioic acid, Pro-Ala-Phe, and 3-hydroxycinnamic acid (Figure 3F and Figure S1F).

### 2.4. Cluster Analysis of Different Polygonati Rhizoma Samples

**Figure 4:**
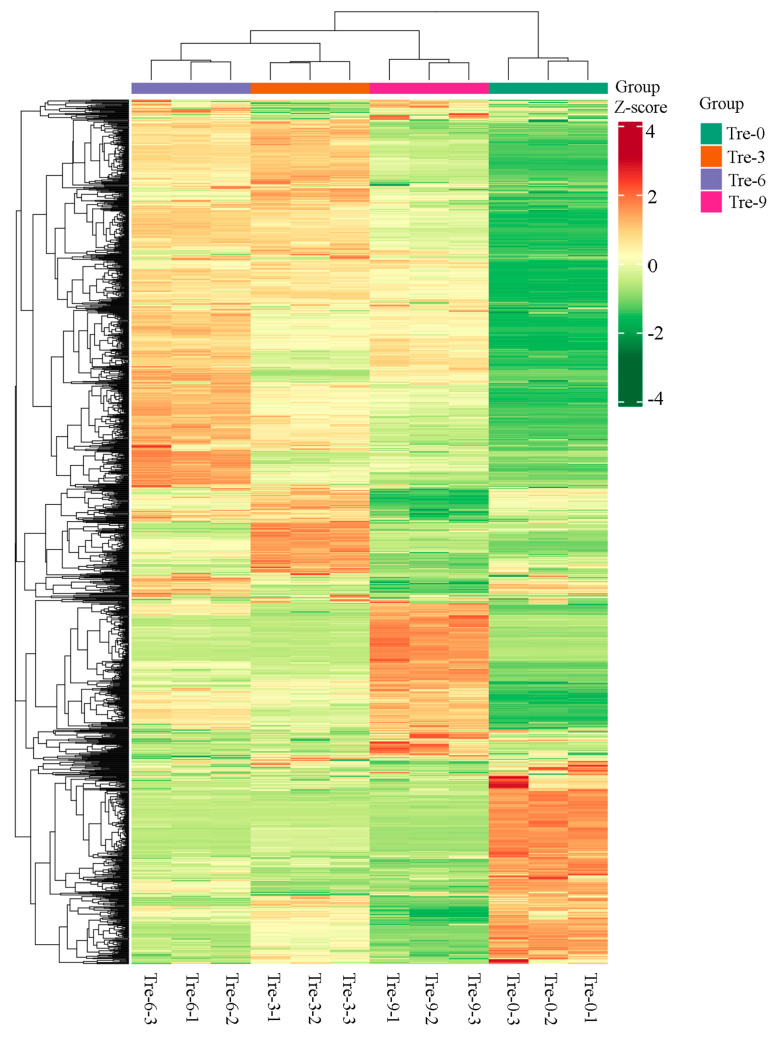
Heatmap visualization of metabolites in four groups of Polygonati Rhizome samples.

In relation to four groups of Polygonati Rhizoma samples undergoing different steaming–drying processes, metabolite compositions differed, with 1330, 1365, 1363, and 1360 metabolites in ‘Tre-0’, ‘Tre-3’, ‘Tre-6’, and ‘Tre-9’ samples, respectively (Table S1). Based on the metabolic profiles, samples ‘Tre-3’ and ‘Tre-6’ were firstly clustered together and then grouped with ‘Tre-9’ samples, while ‘Tre-0’ was clustered alone (Figure 4). In particular, the accumulation of secondary metabolites displayed clear phenotypic variation in different Rhizoma Polygonati samples. Positive correlation existed between signal intensities produced from mass spectrum and abundance of metabolites. According to the sample-specific accumulation patterns, metabolites could be clearly grouped into three main clusters with four sub-clusters. Multivariate statistics was carried out to explore the differential accumulation of metabolites in different Rhizoma Polygonati samples (Figure S2). According to the Pearson correlation coefficients among different samples, biological replicates in each Rhizoma Polygonati sample had high correlation, as the values ranged from 0.38 (‘Tre-0’ vs. ‘Tre-9’) to 0.92 (‘Tre-3’ vs. ‘Tre-6’).

## 3. Discussion

*P. cyrtonema*, a perennial edible plant widely distributed in China, has being served as a food and traditional Chinese medicine for over 2000 years [22]. Flavonoids, saponins, polysaccharides, and alkaloids have all been isolated from *P. cyrtonema* rhizomes [23,24]. The processing methods, such as steaming, drying, and alcohol evaporation, can change the chemical composition of *P*. *cyrtonema* medicinal section [25]. Compounds in Polygonati Rhizome exhibit diverse pharmacological effects, including anti-aging, anti-fatigue, anti-inflammatory, antioxidant, and sleep-enhancing effects, as well as therapeutic potential for osteoporosis and age-related diseases [26]. In this research, alkaloids, flavonoids, lignans, coumarins, lipids, quinones, steroids, terpenoids, amino acids, vitamins, saccharides, nucleotides, organic acids, and phenolic acids, as well as chromone, were all detected based on UPLC-ESI-MS/MS, and might exert health-promoting activities through comprehensive regulation.

Compared with non-processed Polygonati Rhizome, the processed samples were darker, softer, and sweeter; for this reason, they are usually used in traditional medicine and the food industry without causing throat irritation and other side effects [8]. During the repeated steaming–drying processes, the contents of metabolites in Polygonati Rhizome varied, including alkaloids, amino acids and derivatives, flavones, flavonols, glycerol ester, LPE, nucleotides, organic acids, and phenolic acids, which were mainly enriched into pathways of “Metabolic pathways”, “Biosynthesis of cofactors”, “Biosynthesis of secondary metabolites”, “ABC transporters”, “Biosynthesis of amino acids”, “Nucleotide metabolism”, “Flavonoid biosynthesis”, and “Purine metabolism”. In particular, 39 metabolites newly occurred during repeated steaming–drying, mainly alkaloids, amino acid derivatives, flavonoids, lignans and coumarins, short-chain fatty acids, nucleotide derivatives, organic acids, phenolic acids, and terpenoids, which might account for a lot of the variations in the appearance and potential medicinal properties of Polygonati Rhizome.

Flavonoids, widespread plant secondary metabolites, are vital for pigmentation, pharmaceuticals activities, mediating plant–microbe interactions, enhancing plant resistance to pests and diseases, improving plant responses to environmental stresses, and protecting plants from ultraviolet-B damage [27,28,29,30]. Up to now, 20 bioactive flavonoids, including homoisoflavones, isoflavones, chalcones, dihydroflavones, rosandalanes, and flavones, have been isolated from Polygonati Rhizoma [23,31]. In particular, chalcones, dihydroflavones, and rosandalane have been extracted from *P. kingianum*; homoisoflavones and flavones have been mainly isolated from *P. sibiricum* [32,33]. In this research, 167 flavonoids were identified from *P*. *cyrtonema* rhizomes, mainly including chalcones, flavanols, flavanonols, flavones, flavonols, and isoflavones, while the contents were upregulated during repeated steaming and drying. As an example, quercetin possesses significant antioxidant, anti-inflammatory, anti-amyloid, anti-aging, anticancer, antioxidant, and antimicrobial properties [34,35]. In particular, quercetin could inhibit the production of IL-6, IL-1β, and TNF-α to improve pathological changes of tissue [36,37,38]. In relation to Polygonati Rhizome’s remarkable anti-inflammatory and immune-enhancing activity, five derivatives of quercetin, including 3,4′-dimethylquercetin, quercetin-7-*O*-glucoside, quercetin-3-*O*-rutinoside, 6-C-methylquercetin-3-*O*-rutinoside, and quercetin-3-*O*-robinobioside, were detected in both treated and untreated samples. All these flavonoids need to be further isolated for bioactive analysis. 

*P. cyrtonema* polysaccharides, 8.5 × 10^3^–42,400 × 10^3^ Da in triple-helical structures, mainly included glucose, mannose, galactose, arabinose, fructose, rhamnose, ribose, xylose, glucuronic acid, and galacturonic acid, as well as branched homogalactan and galactomannans [39,40]. Besides the β-d-fructofuranosyl, α-d-galactopyranose, α-d-glucopyranose, and *O*-acetyl group, *P. cyrtonema* polysaccharides also contain branched fructan cores with (2→6)-linked-D-Fruf residues every three (2→1)-linked-D-Fruf residues [40]. The surface of the polysaccharides became much tighter during the repeated steaming–drying process [41]. The amount of Rhizoma Polygonati polysaccharides was positively correlated with the expression of β-fructofuranosidase, fructokinase, mannose-1-phosphate guanylyltransferase, and UDP-glucose 6-dehydrogenase genes, which were assigned to a starch and sucrose metabolism pathway [1]. To our discovery, monosaccharide, disaccharide, trisaccharide, tetrasaccharide, and pentasaccharide were all present in Polygonati Rhizome. In particular, phosphorylated derivatives, sugar acid, and sugar alcohols were the main modification forms, which might endow Polygonati Rhizome with significant biological activity. The “Metabolic pathways” and “Biosynthesis of secondary metabolites” were typical pathways for our Rhizoma Polygonati samples.

In total, 77 steroidal saponins, possessing hypoglycemic, anti-inflammatory, immunoregulation, and anti-diabetic properties, have been isolated from the ethanol and methanol extracts of Rhizoma Polygonati [42,43,44]. A list of 12 triterpenoid saponins have been successfully isolated from both ethanol and methanol extracts of rhizoma of *P. sbiricum* and *P. kingianum* [43]. Steroidal saponins isolated from Rhizoma Polygonati are commonly bidesmosides, having a sugar chain attached at C-3 position and a D-glucose residue attached at C-26 position [43]. In this research, 13 well-identified steroidal saponins were detected in the untreated rhizome samples, while one triterpenoid saponin (isorhamnetin-3-*O*-rutinoside) was newly detected in *P. cyrtonema* rhizoma.

As wild *P. cyrtonema* plants are on the brink of extinction, industrial-scale cultivation and production of *P. cyrtonema* has been attempted in recent years. Due to multiple health-promoting activities, the demand for Polygonati Rhizome has increased dramatically year by year. Nowadays, over 365 kinds of commercial food products derived from Polygonati Rhizome are on the market [45,46]. Therefore, our profiles of bioactive metabolites will benefit the manual processing of Polygonati Rhizome. The accumulation of total secondary metabolites displayed a clear compound variation, and samples of ‘Tre-3’ and ‘Tre-6’ were clustered together and then with ‘Tre-9’ samples, inferring similar metabolite composition. A total of 285 compounds had the highest contents in ‘Tre-9’ samples, especially flavones. This research will be greatly helpful in providing a critical scientific basis for genetic improvement and manual processing of Polygonati Rhizome. Further studies are still greatly required to elucidate the full spectrum of cellular mechanisms, which might broaden the application of Rhizoma Polygonati in the functional food field.

## 4. Materials and Methods

### 4.1. Material Preparation

Fresh *P. cyrtonema* rhizomes were collected from the Dabie Mountains (30.215° N, 115.425° E, 350–670 m) (Huanggang, Hebei Province, China) and subjected to a contemporary method involving initial washing to remove soil, subsequent removal of fibrous roots, and air drying until 30% water content. The obtained *P. cyrtonema* rhizomes were equally divided into four groups. One group served as the control (Tre-0), while the other three groups were subjected to processes of three cycles of steaming–drying (Tre-3), six cycles of steaming–drying (Tre-6), and nine cycles of steaming–drying (Tre-9), respectively (Figure 1). In particular, samples were steamed at 120 °C for 2 h and then dried at 50 °C for 6 h in each cycle of steaming and drying to achieve the desired characteristics. Then, these samples were separately cut into slices and lyophilized with liquid nitrogen.

### 4.2. Extraction and LC-MS Analysis

The freeze-dried *P. cyrtonema* rhizomes were ground with a mixer mill (MM 400, Retsch, Haan, Germany) for 1.5 min at 30 Hz. Then, 50 mg mass of powder was extracted overnight with 1.2 mL of 70% aqueous methanol at 4 °C. Following centrifugation (12,000× *g*, 3 min), extracts were filtered and analyzed with the UPLC-ESI-MS/MS system (Ultra Performance Liquid Chromatography, ExionLC™ AD, http://sciex.com.cn/ accesssed on 20 October 2024; MS, Applied Biosystems 4500 Q TRAP, http://www.appliedbiosystems.com.cn/ accesssed on 20 October 2024). In particular, liquid chromatographic analysis was carried out with an Agilent SB-C18 (1.8 μm, 2.1 mm × 100 mm, Agilent Technologies, Santa Clara, CA, USA) at 40 °C column temperature. A volume of 2 μL aqueous methanol was injected, and the flow rate was set as 0.35 mL/min. The solvent system contained solvent A (water with 0.1% formic acid) and solvent B (acetonitrile with 0.1% formic acid). A gradient program was employed (solvent A/B: 100:0 *V*/*V* at 0 min, 5:95 *V*/*V* at 10.0 min, 5:95 *V*/*V* at 11.0 min, 95:5 *V*/*V* at 11.1 min, and finally 95:5 *V*/*V* at 14.0 min) [27].

Effluent was connected to an ESI-triple quadrupole-linear ion trap (Q TRAP)-MS. ESI source operation parameters were set as follows: source temperature of 550 °C; ion spray voltage (IS) 5500 V; 50 psi of ion source gas I; 60 psi of gas II; 25 psi of curtain gas; and collision gas being high. QQQ scans were obtained through multiple reaction monitoring (MRM) experiments with collision gas (nitrogen) being set to moderate. The declustering potential and collision energy assessments for individual MRM transitions were performed through optimization. According to the eluted metabolites for each period, a certain set of MRM transitions was monitored. The data record was controlled with Mass Hunter software (Qualitative Analysis B.07.00) (Agilent Technologies, Madrid, Spain). In particular, Analyst 1.6.3 software (AB Sciex, Framingham, MA, USA) was used for data acquisition, peak integration, and calculations.

### 4.3. Identification and Analysis of Metabolites

Quantification of the metabolites was carried out in MRM mode. In addition, qualitative analysis of the metabolites was carried out according to the MVDB V3.0 Database of Wuhan Metware Biotechnology Co., Ltd. (Wuhan, China). Compounds were identified through comparing accurate *m*/*z* values, retention time (RT), and fragmentation patterns with corresponding standards. Regarding metabolites without corresponding standards, fragmentation patterns were used to query the MS^2^ spectral data or to search the MassBank database. Metabolite data were firstly log2 transformed for improving normality, and then subjected to principal component analysis (PCA) and hierarchical cluster analysis (HCA). In particular, the ‘heatmap.2’ function in ‘gplot’ R-package version 3.2.0 (http://cran.r-project.org/web/packages/gplots/index.html accesssed on 20 October 2024) was utilized. VIP (variable importance in projection) values and FC (fold change) scores were used to identify differentially accumulated metabolites between different samples. The values VIP > 1 and FC ≥ 2 or FC ≤ 0.5 were set as standard. One-way ANOVA (*p* ≤ 0.05) was also carried out. The SIMCA-P 14.0 software package was used for the orthogonal partial least squares discriminant analysis (OPLS-DA).

## Figures and Tables

**Table 1:** Metabolite composition in four different Polygonati Rhizoma samples.

Class (Numbers of Metabolites)	Sub-Class	Numbers in Different Samples				
		Tre-0	Tre-3	Tre-6	Tre-9	Total
Alkaloids (149)	Alkaloids	69	71	71	71	71
	Benzylphenylethylamine alkaloids	1	1	1	1	1
	Isoquinoline alkaloids	2	2	2	2	2
	Phenolamine	36	36	36	35	36
	Piperidine alkaloids	3	3	3	3	3
	Plumerane	20	20	20	20	20
	Pyridine alkaloids	4	4	4	4	4
	Pyrrole alkaloids	4	4	4	4	4
	Quinoline alkaloids	6	6	6	6	6
	Tropan alkaloids	2	2	2	2	2
Flavonoids (167)	Chalcones	9	9	9	8	9
	Flavanols	4	4	4	4	4
	Flavanones	16	16	16	16	16
	Flavanonols	2	2	2	2	2
	Flavones	58	62	62	62	63
	Flavonols	14	14	14	13	15
	Isoflavones	9	9	8	9	9
	Other flavonoids	48	49	49	49	49
Lignans and coumarins (46)	Coumarins	11	11	11	11	11
	Lignans	33	35	35	35	35
Lipids (158)	Free fatty acids	79	80	80	80	80
	Glycerol ester	7	10	10	10	10
	LPC	29	29	29	29	29
	LPE	29	31	31	31	31
	PC	1	1	1	1	1
	Sphingolipids	7	7	7	7	7
Phenolic acids (181)	Phenolic acids	176	181	181	178	181
Quinones (14)	Anthraquinone	9	9	9	9	9
	Quinones	5	5	5	5	5
Steroids (19)	Steroid	6	6	6	6	6
	Steroidal saponins	13	13	13	13	13
Terpenoids (37)	Ditepenoids	15	15	15	15	15
	Monoterpenoids	10	10	10	10	10
	Terpene	4	4	4	4	4
	Triterpene	7	7	7	7	7
	Triterpene saponin	0	1	1	1	1
Nucleotides and derivatives (71)	Nucleotides and derivatives	68	71	71	71	71
Organic acids (115)	Organic acids	113	114	113	115	115
Amino acids and derivatives (248)	Amino acids and derivatives	242	248	247	248	248
Others (164)	Alcohol compounds	5	5	5	5	5
	Aldehyde compounds	8	8	8	8	8
	Chromone	2	2	2	2	2
	Ketone compounds	13	14	14	14	14
	Others	41	44	44	44	44
	Saccharides	73	73	74	73	74
	Vitamin	17	17	17	17	17
	Total	1330	1365	1363	1360	1369

**Table 2:** Modification of amino acids identified in *P. cyrtonema* rhizomes.

No.	Modification	Corresponding Amino Acids
1	Acetylation	Arg, Glu, Asp Thr, Gln, Ala, Lys, Phe, Leu, Asp, Tyr, Ser
2	Cyclo-	Tyr, Ala, Val, Pro, Phe, Leu, Glu, Gly, Ser
3	Hydroxylation	Phe, Tyr, Pro, Glu, Leu, Met, His
4	Monomethylation	Glu, Pro,Arg, Cys,His, Met
5	Dimethylation	Lys, Arg,Gly
6	Trimethylation	Lys
7	Homo-	Ser, Ala, Pro, Arg, Cys
8	Oxo-	Pro, Tyr
9	Glycosidation	Asp, Glu
10	Adenosylation	Met, Cys
11	Esterification	Arg
12	Ethylation	Gly
13	Benzylation	Ser
14	Propylation	Pro
15	Ribosylation	Cys
16	Propionylation	Gly
17	Propenylation	Cys
18	Allosterism	Leu
19	Cyano-	Ala
20	Acylation	Gly
21	Nitro-	Tyr
22	Phosphorylation	Tyr

**Table 3:** Saccharides identified in *P. cyrtonema* rhizomes.

Types	Compounds	Molecular Weight (Da)	Formula	Modification
monosaccharide	Dihydroxyacetone phosphate	169.998	C_3_H_7_O_6_P	phosphorylation
	D-threonic acid	136.0372	C_4_H_8_O_5_	oxidation
	3-dehydro-L-threonic acid	134.0215	C_4_H_6_O_5_	oxidation
	D-erythrose-4-phosphate	200.0086	C_4_H_9_O_7_P	phosphorylation
	D-threose	120.0423	C_4_H_8_O_4_	
	D-xylonic acid	166.0477	C_5_H_10_O_6_	oxidation
	L-xylose	150.0528	C_5_H_10_O_5_	_
	D-arabinose	150.0528	C_5_H_10_O_5_	_
	D-arabinono-1,4-lactone	148.0372	C_5_H_8_O_5_	lactonization
	D-fructose-1,6-biphosphate	339.996	C_6_H_14_O_12_P_2_	phosphorylation
	D-glucosamine 1-phosphate	259.0457	C_6_H_14_NO_8_P	phosphorylation
	D-fructose 6-phosphate	260.0297	C_6_H_13_O_9_P	phosphorylation
	Gluconic acid	196.0583	C_6_H_12_O_7_	oxidation
	D-glucoronic acid	194.0427	C_6_H_10_O_7_	oxidation
	D-glucose 6-phosphate	260.0297	C_6_H_13_O_9_P	phosphorylation
	D-glucurono-6,3-lactone	176.0321	C_6_H_8_O_6_	lactonization
	D-glucose	180.0634	C_6_H_12_O_6_	_
	D-glucose 1,6-bisphosphate	339.996	C_6_H_14_O_12_P_2_	phosphorylation
	D-glucosamine	259.0457	C_6_H_13_NO_5_	amination
	Glucose-1-phosphate	260.0297	C_6_H_13_O_9_P	phosphorylation
	D-glucono-1,5-lactone	178.0477	C_6_H_10_O_6_	lactonization
	Glucaric acid-1-phosphate	290.0039	C_6_H_11_PO_11_	phosphorylation
	1,6-anhydro-β-d-glucose	162.0528	C_6_H_10_O_5_	dehydration
	D-sorbitol	182.079	C_6_H_14_O_6_	reduction
	sorbose	180.0634	C_6_H_12_O_6_	_
	Sorbitol-6-phosphate	262.0454	C_6_H_15_O_9_P	phosphorylation
	D-mannitol	182.079	C_6_H_14_O_6_	reduction
	D-mannose	180.0634	C_6_H_12_O_6_	_
	1,5-anhydro-d-glucitol	164.0685	C_6_H_12_O_5_	dehydration, reduction
	D-fucose	164.0685	C_6_H_12_O_5_	_
	L-fucitol	166.0841	C_6_H_14_O_5_	reduction
	D-galacturonic acid	194.0427	C_6_H_10_O_7_	oxidation
	Dulcitol	182.079	C_6_H_14_O_6_	reduction
	D-galactose	180.0634	C_6_H_12_O_6_	_
	D-galactaric acid	210.0376	C_6_H_10_O_8_	oxidation
	D-saccharic acid	210.0376	C_6_H_10_O_8_	oxidation
	Rhamnose	164.0685	C_6_H_12_O_5_	_
	L-gulono-1,4-lactone	178.0477	C_6_H_10_O_6_	-
	Allitol	182.079	C_6_H_14_O_6_	reduction
	Inositol	180.0634	C_6_H_12_O_6_	_
	D-sedoheptuiose 7-phosphate	290.0403	C_7_H_15_O_10_P	phosphorylation
	Sedoheptulose	210.074	C_7_H_14_O_7_	_
	D-pinitol	194.079	C_7_H_14_O_6_	reduction
	N-Acetyl-d-galactosamine	221.0899	C_8_H_15_NO_6_	amination
	1-(sn-Glycero-3-phospho)-1D-myo-inositol	334.0665	C_9_H_19_O_11_P	reduction
	Glucopyranose 6-hydroxydecanoate	350.1941	C_16_H_30_O_8_	esterification
disaccharide	Sucrose-6-phosphate	422.0825	C_12_H_23_O_14_P	phosphorylation
	D-sucrose	342.1162	C_12_H_22_O_11_	-
	Galactinol	342.1162	C_12_H_22_O_11_	reduction
	D-cellobiose	342.1162	C_12_H_22_O_11_	-
	D-trehalose	342.1162	C_12_H_22_O_11_	-
	Maltitol	344.1319	C_12_H_24_O_11_	-
	Isomaltulose	342.1162	C_12_H_22_O_11_	-
	D-maltose	342.1162	C_12_H_22_O_11_	-
	Lactobiose	342.1162	C_12_H_22_O_11_	-
	Digalacturonate	370.0747	C_12_H_18_O_13_	-
	Melibiose	342.1162	C_12_H_22_O_11_	-
	Rutinose	326.1213	C_12_H_22_O_10_	-
	Trehalose 6-phosphate	422.0825	C_12_H_23_O_14_P	phosphorylation
	beta-d-Galp-(1->3)-d-GalpNAc	383.1428	C_14_H_25_NO_11_	
trisaccharide	Planteose	504.169	C_18_H_32_O_16_	-
	Maltotriose	504.169	C_18_H_32_O_16_	-
	Manninotriose	504.169	C_18_H_32_O_16_	-
	D-Panose	504.169	C_18_H_32_O_16_	-
	Solatriose	488.1741	C_18_H_32_O_15_	-
	Gentianose	504.1690	C_18_H_32_O_16_	-
	Raffinose	504.169	C_18_H_32_O_16_	-
	D-melezitose	504.169	C_18_H_32_O_16_	-
	GalNAc1-4[Fuc1-3]GlcNAcSp	639.2599	C_24_H_41_N_5_O_15_	-
Tetrasaccharide	D-maltotetraose	666.2219	C_24_H_42_O_21_	_
	Stachyose	666.2219	C_24_H_42_O_21_	-
	DMelezitose O-rhamnoside	650.2269	C_24_H_42_O_20_	_
	Nystose	666.2219	C_24_H_42_O_21_	-
Pentasaccharide	Verbascose	828.2747	C_30_H_52_O_26_	-

**Table 4:** Statistical information of metabolites with the highest contents in different samples.

Class	Sub-Class	Numbers of Metabolites with the Highest Contents				Total
		Tre-0	Tre-3	Tre-6	Tre-9	
Alkaloids (149)	Alkaloids	25	22	15	9	71
	Benzylphenylethylamine alkaloids	1	0	0	0	1
	Isoquinoline alkaloids	2	0	0	0	2
	Phenolamine	16	13	2	5	36
	Piperidine alkaloids	2	0	1	0	3
	Plumerane	8	3	7	2	20
	Pyridine alkaloids	2	1	1	0	4
	Pyrrole alkaloids	0	1	3	0	4
	Quinoline alkaloids	3	1	2	0	6
	Tropan alkaloids	0	1	1	0	2
Amino acids and derivatives (248)	Amino acids and derivatives	67	53	58	70	248
Flavonoids (167)	Chalcones	4	3	2	0	9
	Flavanols	1	2	0	1	4
	Flavanones	2	9	5	0	16
	Flavanonols	2	0	0	0	2
	Flavones	10	9	19	25	63
	Flavonols	4	1	6	4	15
	Isoflavones	4	0	0	5	9
	Other Flavonoids	22	17	5	5	49
Lignans and coumarins (46)	Coumarins	2	3	6		11
	Lignans	4	9	17	5	35
Lipids (158)	Free fatty acids	1	22	51	6	80
	Glycerol ester	1	0	7	2	10
	LPC	1	6	23	0	29
	LPE	0	19	11	0	31
	PC	1	0	0	0	1
	Sphingolipids	4	2	1	0	7
Nucleotides and derivatives (71)	Nucleotides and derivatives	12	31	14	14	71
Organic acids (115)	Organic acids	24	11	46	34	115
Others (164)	Alcohol compounds	0	2	2	1	5
	Aldehyde compounds	4	0	4	0	8
	Chromone	2	0	0	0	2
	Ketone compounds	5	4	5	0	14
	Others	7	16	19	2	44
	Saccharides	12	9	19	34	74
	Vitamin	2	7	5	3	17
Phenolic acids (181)	Phenolic acids	39	59	39	44	181
Quinones (14)	Anthraquinone	0	2	5	2	9
	Quinones	2	1	2	0	5
Steroids (19)	Steroid	2	3	0	1	6
	Steroidal saponins	9	2	2	0	13
Terpenoids (37)	Ditepenoids	0	1	8	6	15
	Monoterpenoids	4	4	0	2	10
	Terpene	1	0	3	0	4
	Triterpene	0	3	1	3	7
	Triterpene saponin	0	0	1	0	1

## Data Availability

Data are contained within the article and Supplementary Materials.

## Supplementary Materials

The following supporting information can be downloaded at: https://www.mdpi.com/article/10.3390/molecules30091923/s1, Figure S1: KEGG pathway enrichment analysis of differentially accumulated metabolites: (A) ‘Tre-0’ vs. ‘Tre-3’; (B) ‘Tre-0’ vs. ‘Tre-6’; (C) ‘Tre-0’ vs. ‘Tre-9’; (D) ‘Tre-3’ vs. ‘Tre-6’; (E) ‘Tre-3’ vs. ‘Tre-9’; (F) ‘Tre-6’ vs. ‘Tre-9’; Figure S2: composition correlation analysis among different samples based on Pearson’s correlation coefficient; Table S1: Information of metabolites identified in *P. cyrtonema* rhizomes; Table S2: Vitamins identified in *P. cyrtonema* rhizomes; Table S3: Trends of metabolite content in four different types of Rhizoma Polygonati; Table S4: Representative metabolites of six types with different variation trends; Table S5: Representative metabolites with very low content in any samples; Table S6: Information of secondary metabolites with highest contents in different samples; Table S7: The differential accumulation metabolites between different samples; Table S8: Statistical information of differentially accumulated metabolites between different sample pairs; Table S9: The enriched KEGG pathways of differentially accumulated metabolites between different samples.
