# Supplementary material for: Dynamic Variation of Secondary Metabolites from Polygonatum cyrtonema Hua Rhizomes During Repeated Steaming–Drying Processes
Source: Molecules. 2025 Apr 25;30(9):1923. doi: 10.3390/molecules30091923 (PMC12073103; doi:10.3390/molecules30091923)
Supplement: Supplementary file 1 [file molecules-30-01923-s001.zip › Figure caption.pdf]

Figure S1. KEGG pathway enrichment analysis of differentially accumulated metabolites: (A) ‘Tre-0’ vs ‘Tre-3’; (B) ‘Tre-0’ vs ‘Tre-6’; (C) ‘Tre-0’ vs ‘Tre-9’; (D) ‘Tre-3’ vs ‘Tre-6’; (E) ‘Tre-3’ vs ‘Tre-9’; (F) ‘Tre-6’ vs ‘Tre-9’.

Figure S2. Composition correlation analysis among different samples based on Pearson’s correlation coefficient.
