# Supplementary material for: Dynamic Variation of Secondary Metabolites from Polygonatum cyrtonema Hua Rhizomes During Repeated Steaming–Drying Processes
Source: Molecules. 2025 Apr 25;30(9):1923. doi: 10.3390/molecules30091923 (PMC12073103; doi:10.3390/molecules30091923)
Supplement: Supplementary file 1 [file molecules-30-01923-s001.zip › Table S2 Vitamins identified in P. cyrtonema rhizomes.pdf]

Table S2. Vitamins identified in *P. cyrtonema rhizomes.*

**Types**	**Dissolution Properties**	**Compounds**	**Molecular Weight (Da)**	**Formula**
Vitamin E	fat-soluble	Delta-Tocopherol	4.02E+02	$C_{27}H_{46}O_2$
Vitamin K1	fat-soluble	Phylloquinone	4.50E+02	$C_{31}H_{46}O_2$
Vitamin K2	fat-soluble	Menatetrenone	4.44E+02	$C_{31}H_{40}O_2$
Vitamin C	water-soluble	Erythorbic Acid	1.76E+02	$C_6H_8O_6$
		L-Ascorbic acid	1.76E+02	$C_6H_8O_6$
Vitamin B2	water-soluble	Riboflavin	3.76E+02	$C_{17}H_{20}N_4O_6$
Vitamin B3	water-soluble	Nicotinamide	1.22E+02	$C_6H_6N_2O$
		Nicotinic acid	1.23E+02	$C_6H_5NO_2$
		Nicotinate D-ribonucleoside	2.56E+02	$C_{11}H_{14}NO_6^+$
		Isonicotinic acid	1.23E+02	$C_6H_5NO_2$
Vitamin B5	water-soluble	D-Pantothenic Acid	2.19E+02	$C_9H_{17}NO_5$
Vitamin B6	water-soluble	Pyridoxal	1.67E+02	$C_8H_9NO_3$
		4-Pyridoxic acid	1.83E+02	$C_8H_9NO_4$
		4-Pyridoxic acid-O-glucoside	3.45E+02	$C_{14}H_{19}NO_9$
		Pyridoxine	1.69E+02	$C_8H_{11}NO_3$
		Pyridoxine-5'-O-glucoside	3.31E+02	$C_{14}H_{21}NO_8$
Vitamin B13	water-soluble	Orotic acid	1.56E+02	$C_5H_4N_2O_4$
