# Supplementary material for: Dynamic Variation of Secondary Metabolites from Polygonatum cyrtonema Hua Rhizomes During Repeated Steaming–Drying Processes
Source: Molecules. 2025 Apr 25;30(9):1923. doi: 10.3390/molecules30091923 (PMC12073103; doi:10.3390/molecules30091923)
Supplement: Supplementary file 1 [file molecules-30-01923-s001.zip › Table S8 Statistical information of differentially accumulated metabolites between different sample pairs..pdf]

Table S8. Statistical information of differentially accumulated metabolites between different sample pairs.

Class	Sub-class	Tre-0 vs Tre-3		Tre-0 vs Tre-6		Tre-0 vs Tre-9		Tre-3 vs Tre-6		Tre-3 vs Tre-9		Tre-6 vs Tre-9	
		up	down	up	down	up	down	up	down	up	down	up	down
Alkaloids	Alkaloids	22	13	23	14	21	20	6	6	6	19	1	11
	Tropan alkaloids												
	Pyrrole alkaloids	1		1		1							
	Benzylphenylethylamine alkaloids		1		1		1						
	Isoquinoline alkaloids		2		2		2		1		2		2
	Phenolamine	9	5	10	13	9	16	2	11	3	24		9
	Piperidine alkaloids		2		1		2						
	Plumerane	9	7	10	7	9	8	1	1	2	10	1	9
	Pyridine alkaloids	2	2	2	2	2	2						1
	Quinoline alkaloids	2	3	1	3	1	3				3		2
	Tropan alkaloids	1		1							1		
	Total	46	35	48	43	43	54	9	19	11	59	2	34
Amino acids and derivatives	–	123	41	121	42	123	62	27	23	40	58	32	48
Flavonoids	Chalcones	4	3	2	3	2	5		4		4		3
	Flavanols	2							1				
	Flavanones	6	1	6	2	1	2	5	5		7		7
	Flavanonols				1		1		1		1		
	Flavones	21	4	28	4	27	11	16	10	25	14	20	17
	Flavonols	4	3	7	3	2	3	5	1	2	2	1	7
	Isoflavones	1		1	1	3	1		1	2		3	
	Other flavonoids	17	8	14	16	14	18	1	19	2	32		13
	Total	55	19	58	30	49	41	27	42	31	60	24	47
Lignans and Coumarins	Coumarins	5	1	5	2	4	2	1			2		2
	Lignans	21	2	21	3	15	5	3	3	3	13	4	7

	Total	26	3	26	5	19	7	4	3	3	15	4	9
Lipids	Free fatty acids	59		58		49					7		13
	Glycerol ester	6	1	7		6	1						3
	LPC	29		29		29		1			7		5
	LPE	31		31		31			1		17		15
	PC		1					1		1			
	Sphingolipids	1		1		1	2				3		2
	Total	126	2	126		116	3	2	1	1	34		38
Nucleotides and derivatives	_	49	9	47	7	46	10	10	4	8	24	6	24
Organic acids	_	52	11	59	10	64	17	18	3	20	3	10	7
Phenolic acids	_	85	21	90	26	85	39	10	15	26	54	21	42
Quinones	Anthraquinone	8		7		8	3		1	1	3	1	4
	Quinones				3				3		3		
Steroids	Steroid	2	1	1	2		1		2		4		1
	Steroidal saponins	2	4	2	8	1	8		3		12		8
	Total	4	5	3	10	1	9		5		16		9
Terpenoids	Ditepenoids	10		10		9		1		1		1	1
	Monoterpenoids	2		1		2	5		1	2	6	2	4
	Terpene	2	1	2	1	1	1				1		1
	Triterpene	1		1		1							
	Triterpene Saponin	1		1		1							
	Total	16	1	15	1	14	6	1	1	3	7	3	6
Others	Alcohol compounds	2		2		2			1		1		1
	Aldehyde compounds	4	2	3	2	3	2		2		3		3
	Ketone compounds	7	1	7	1	6	3	1			4		6
	Others	23	4	21	4	21	8	2	4	2	11	1	11
	Saccharides	27	8	31	2	38	5	12	3	24	2	17	7
	Vitamin	9	1	10	2	8	2	1	1		5		3
	Total	72	16	74	11	78	20	16	11	26	26	18	31
