# Supplementary figures and images for: Dynamic Variation of Secondary Metabolites from Polygonatum cyrtonema Hua Rhizomes During Repeated Steaming–Drying Processes
Source: Molecules. 2025 Apr 25;30(9):1923. doi: 10.3390/molecules30091923 (PMC12073103; doi:10.3390/molecules30091923)

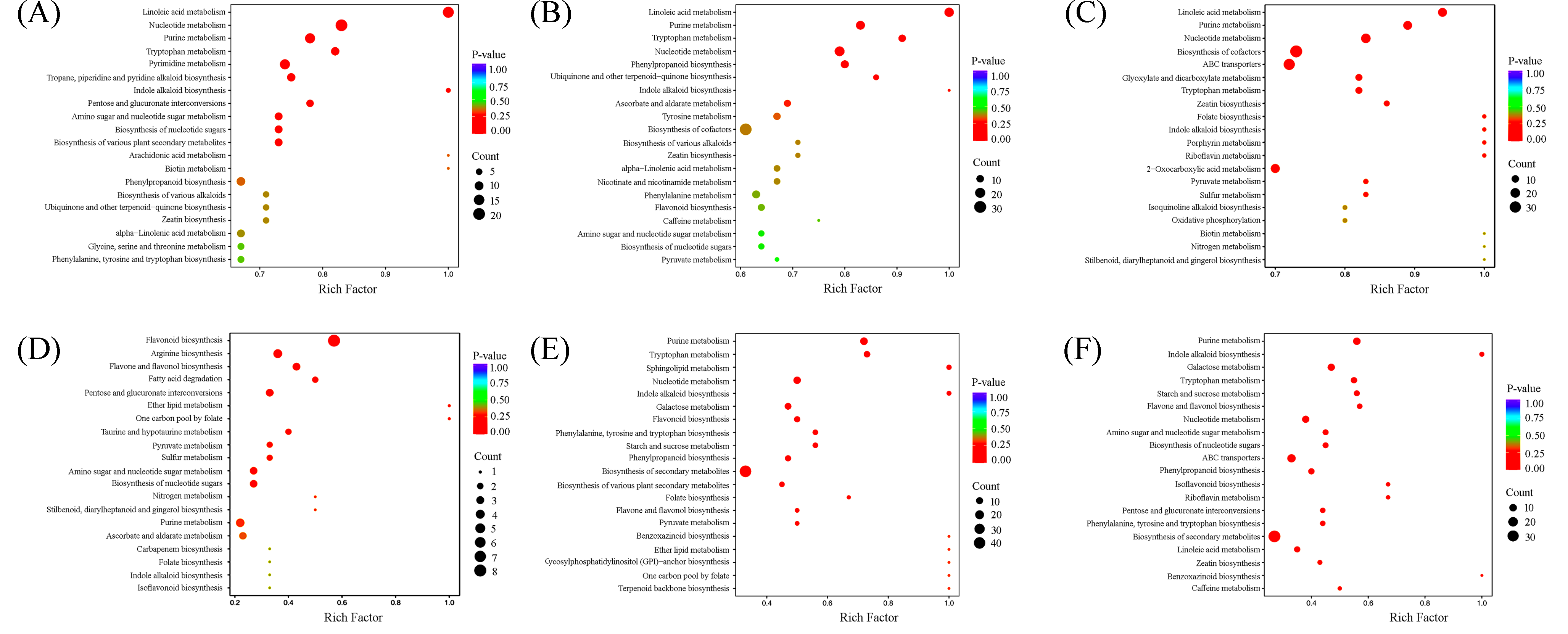

Supplement: Supplementary file 1 [file molecules-30-01923-s001.zip › Figure S1.tif]

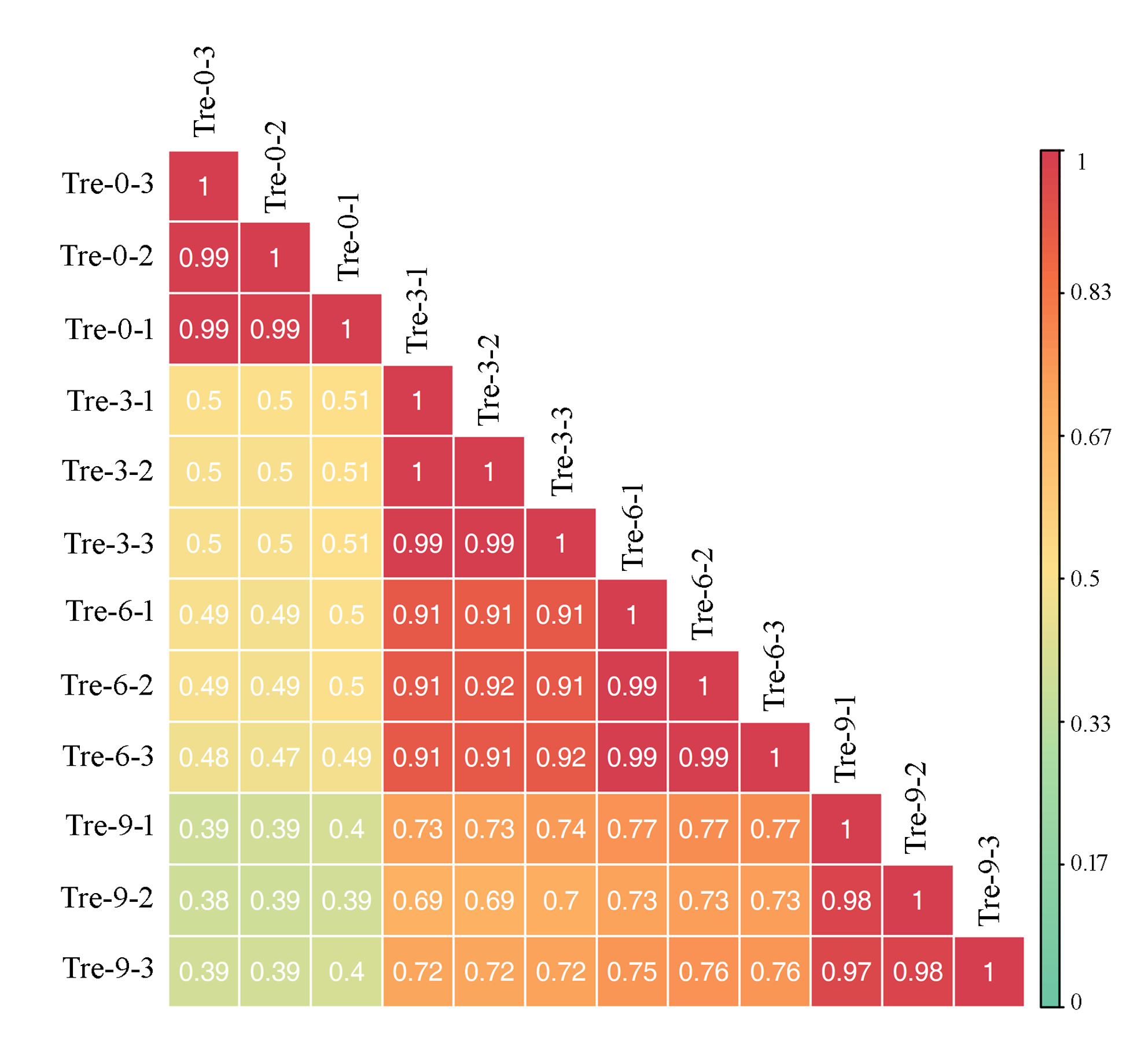

Supplement: Supplementary file 1 [file molecules-30-01923-s001.zip › Figure S2.tif]
